# Temporal Dissociation of Neocortical and Hippocampal Contributions to Mental Time Travel Using Intracranial Recordings in Humans

**DOI:** 10.3389/fncom.2018.00011

**Published:** 2018-02-28

**Authors:** Roey Schurr, Mor Nitzan, Ruth Eliahou, Laurent Spinelli, Margitta Seeck, Olaf Blanke, Shahar Arzy

**Affiliations:** ^1^Neuropsychiatry Lab, Department of Neurology, Hadassah Hebrew University Medical Center, Jerusalem, Israel; ^2^Faculty of Medicine, Hadassah Hebrew University Medical School, Jerusalem, Israel; ^3^Racah Institute of Physics, The Hebrew University of Jerusalem, Jerusalem, Israel; ^4^Department of Radiology, Hadassah Hebrew University Medical Center, Jerusalem, Israel; ^5^Department of Neurology, University Hospital, Geneva, Switzerland; ^6^Laboratory of Cognitive Neuroscience, Swiss Federal Institute of Technology (EPFL), Lausanne, Switzerland

**Keywords:** episodic memory, mental time travel, self-projection, self-reference, hippocampus, lateral temporal, sEEG

## Abstract

In mental time travel (MTT) one is “traveling” back-and-forth in time, remembering, and imagining events. Despite intensive research regarding memory processes in the hippocampus, it was only recently shown that the hippocampus plays an essential role in encoding the temporal order of events remembered, and therefore plays an important role in MTT. Does it also encode the temporal relations of these events to the remembering self? We asked patients undergoing pre-surgical evaluation with depth electrodes penetrating the temporal lobes bilaterally toward the hippocampus to project themselves in time to a past, future, or present time-point, and then make judgments regarding various events. Classification analysis of intracranial evoked potentials revealed clear temporal dissociation in the left hemisphere between lateral-temporal electrodes, activated at ~100–300 ms, and hippocampal electrodes, activated at ~400–600 ms. This dissociation may suggest a division of labor in the temporal lobe during self-projection in time, hinting toward the different roles of the lateral-temporal cortex and the hippocampus in MTT and the temporal organization of the related events with respect to the experiencing self.

## Introduction

A fundamental trait of human cognition is the capacity to engage in “mental time travel” (MTT), to remember past events or imagine possible future ones (Tulving, 1985). When Tulving first presented the concept of MTT, it was proposed as a means of extending and binding together the two more basic functions of episodic memory and episodic future thinking, also known as “prospection” (Schacter and Addis, 2007; Suddendorf and Corballis, 2007; Bar, 2009; Spreng et al., 2009; Schacter et al., 2012). Over the years, the concept of MTT was developed beyond the common neurocognitive basis of past and future thinking to include several different functions (Spreng et al., 2009; Schacter et al., 2012). The process of “scene construction” has been suggested as a key component of MTT, allowing the retrieval of relevant elements from memory and their subsequent binding into a coherent spatial scene (Hassabis et al., 2007a; Maguire and Mullally, 2013). Another process suggested as a fundamental aspect of MTT is self-projection in time, namely the ability to disengage from the immediate environment and mentally “project” oneself to a new “self-location” in time, either in the past or in the future (Buckner and Carroll, 2007; Arzy et al., 2008; Nyberg et al., 2010; Markowitsch and Staniloiu, 2011; Klein, 2013; Kurczek et al., 2015). It is from this “self-location” in time that the individual re-orients herself with respect to different events, in past or future (Arzy et al., 2009a; Peer et al., 2015). To reiterate, MTT comprises of several distinct processes, among them: self-projection to a specific self-location in time, imagination of the relevant event (that is, the act of remembering a past event or of prospecting a future one), and self-orientation with respect to other events (Peer et al., 2014, 2015).

Similarly to the way in which the field of memory research has progressed from focusing on autobiographical memory to the broader notion of MTT and related concepts, the study of their neuroanatomical substrate has also advanced. Whereas, early studies of memory functions focused on the hippocampus, various studies have since established the existence of a large-scale brain network supporting MTT-related processes (Buckner and Carroll, 2007; Hassabis et al., 2007a; Arzy et al., 2009a; Schacter and Addis, 2009; Spreng et al., 2009; Nyberg et al., 2010; Benoit and Schacter, 2015). The key regions of this network include the medial prefrontal, posterior parietal, and lateral temporal cortices, and the medial temporal lobe, including the hippocampus (Addis et al., 2007; Arzy et al., 2009a; Spreng et al., 2009; Rugg and Vilberg, 2013). Notably, although the hippocampus is considered a key region in this “core” network (McNaughton and Morris, 1987; Squire, 1992, 2004; Carpenter and Grossberg, 1993; Moll and Miikkulainen, 1997; Scoville and Milner, 2000; Yonelinas, 2002; Burgess et al., 2007; Bird and Burgess, 2008), its specific involvement in MTT is still debated. For example, while some reported hippocampal involvement in future thinking (Okuda et al., 2003; Hassabis et al., 2007b; Schacter and Addis, 2009), others reported evidence suggesting that future thinking could be independent of the hippocampus (Squire et al., 2010; Hurley et al., 2011).

Moreover, elucidating the differential contributions of the hippocampus and neocortical regions to MTT may have profound implications for the ongoing debate regarding the role of the hippocampus in both memory functions and spatial cognition, including representation of the immediate space, navigation and spatial orientation (O'Keefe and Dostrovsky, 1971; Doeller et al., 2008; Dombeck et al., 2010; Buzsáki and Moser, 2013; Eichenbaum and Cohen, 2014; Hartley et al., 2014). Several attempts have been made to reconcile the role of the hippocampus in memory functions and spatial cognition. The “relational memory theory” suggests that the hippocampus offers a general relational processing mechanism, providing similar computations for the encoding of episodes as sequences of events, and the encoding of routes as sequences of places traversed (Konkel and Cohen, 2009; Eichenbaum and Cohen, 2014). Alternatively, the abovementioned “scene construction theory” asserts that the hippocampus supports episodic memories and imagined future events by facilitating the generation of atemporal scenes, binding together the event's disparate elements into a coherent whole (Maguire and Mullally, 2013). Under this view, the hippocampus is thought to support spatial navigation by virtue of ongoing anticipatory scene construction, giving rise to a continuous representation of the upcoming spatial environment. While different empirical results support both theories, decisive experimental evidence for the role of the hippocampus in MTT is still required.

**Figure 1:**
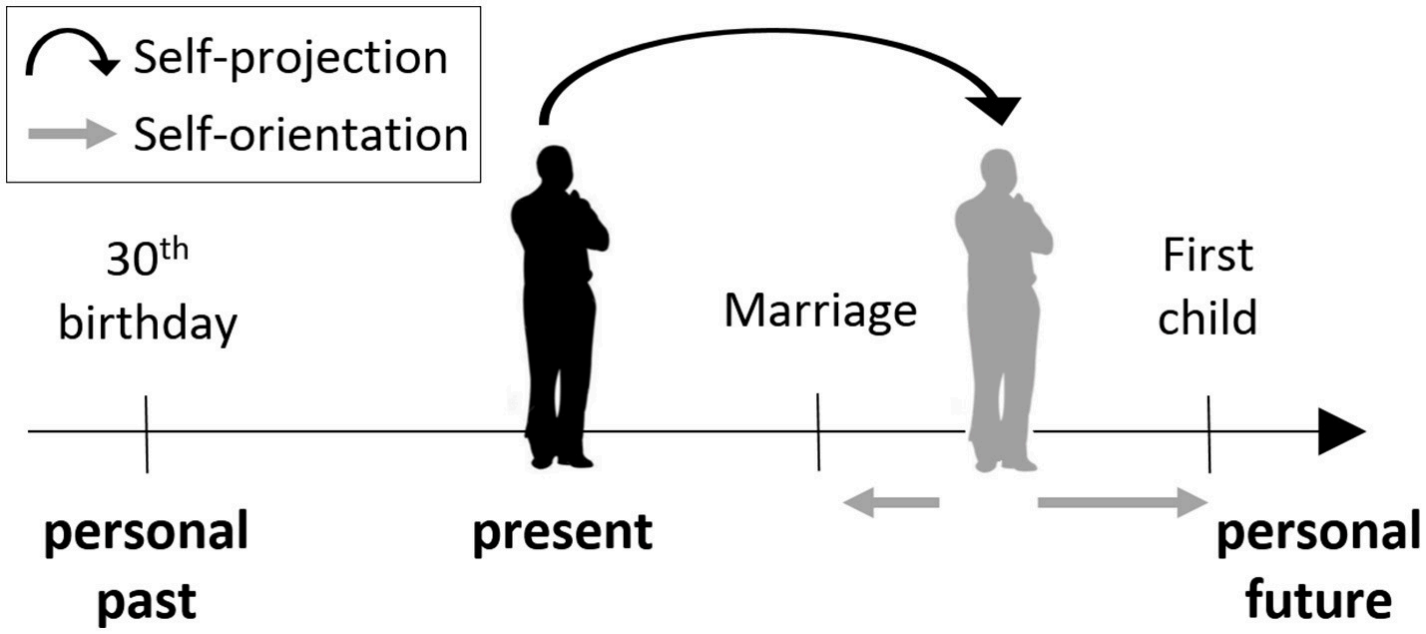
The mental time travel (MTT) task. Participants were asked to “project” themselves to an imagined self-location in the past or future. From this self-location, or from the present one, they were asked to make judgments indicating their orientation with respect to different events, that is, whether the event has already happened or is yet to happen, relative to the participant's location in time.

To investigate the role of the hippocampus in MTT we recorded intracranial evoked potentials (iEPs) in response to an established task of self-projection in time (Arzy et al., 2008, 2009a; Figure 1) in three patients with epilepsy undergoing pre-surgical evaluation. Patients were requested to imagine themselves either in the present self-location in time (“now”) or in another self-location, either 10 years toward the past or toward the future (“then”). It is from this self-location in time that they had to make judgments with respect to different events. For control purposes, iEPs were recorded also when patients performed a spatial task requiring self-projection in space (Arzy et al., 2006). Patients were implanted with bitemporal depth electrodes, penetrating both the hippocampus and the lateral temporal cortex (LTC), a major region in the cortical network involved in MTT (Svoboda et al., 2006; Arzy et al., 2008; Spreng et al., 2009; Benoit and Schacter, 2015; Peer et al., 2015). Such stereo-electroencephalography (sEEG) depth electrodes enable the separation of neocortical and hippocampal activities in both the time and space domains, unlike other neuroimaging methods, with lower spatial or temporal resolution (such as EEG and functional MRI, respectively). This setting enabled us to classify the temporal dynamics of brain activity in the hippocampus and LTC, to better understand the role of these regions in MTT.

## Materials and methods

### Participants

Participants were three right-handed epileptic patients (17, 18, and 40 years old) who suffered from complex partial seizures resistant to pharmacological treatment, with no history of psychiatric or other neurological disorders. In order to localize the seizure onset zone and to dissociate it from essential cortex, intracranial electrodes were implanted. One patient was diagnosed with an epileptic focus in the right temporal pole, one with a left frontal focus, and in one the epileptic focus was found in the left amygdala. Written informed consent was obtained, and the procedures were approved by the Ethical Committee of the University Hospital of Geneva.

### Stimuli and procedures

In the MTT task (Arzy et al., 2008) participants are first asked to imagine themselves either at the present time (“now”), or in another time point (“then”), 10 years in the past or in the future. Participants are then presented with events from personal life (e.g., car license; first child) or non-personal world events (e.g., Challenger explosion; Obama's election), and are asked to indicate whether this event takes place before or after the currently imagined time-point (Figure 1). Thus, participants are requested to mentally “project” themselves in time in order to accomplish the task. Stimuli were designed to be in the range of ±10 years of the imagined time-point, and included events that were chosen from a validated list of common personal life events for the personal items, and from major headline news events for the non-personal items (Arzy et al., 2008, 2009a). Stimuli appeared for 700 ms in the center of a computer screen with an inter-stimulus interval of 2,000 ms as used previously (Arzy et al., 2008). Judgments were given using index and middle fingers of the left and right hand in alternating blocks as a button press on a serial response box. Participants were instructed to respond as quickly and precisely as possible while maintaining a mental image of themselves in the appropriate time-point (“now,” “past,” or “future”). These conditions were performed in different blocks and counterbalanced across participants. Each block included 120 stimuli, equally distributed among four groups appearing in random order: personal-events/world-events × before/after.

As a control task, participants also performed a spatial task involving own-body transformation (Blanke et al., 2005). This task presents participants with a schematic human figure, either facing toward them or away from them, with the figure's right or left hand marked by a ribbon. Participants either responded from their present location (“here”), or were asked to mentally “project” themselves to the location represented by the schematic figure (“there”). It is from this perspective that they made judgments regarding the presented figure (Figure S1; Blanke et al., 2005; Arzy et al., 2006). In the “there” condition, participants were instructed to indicate whether the figure's marked hand is the right or left hand. They were instructed to respond as fast and precise as possible, yet always perform the mental projection of their body before responding. In the “here” condition the same visual stimuli were used, and participants were asked to decide from their habitual location whether the indicated hand was on the right or the left side of the computer screen (Blanke et al., 2005). Stimuli appeared for 300 ms in the center of the computer screen. The interstimulus interval was 2,000 ms. Each block included 120 stimuli, equally distributed among the four conditions, counterbalanced across subjects. Since the analysis is done within-task, an optimal duration for stimulus presentation was chosen separately for each task, based on previous studies.

### Overview of implanted electrodes

**Figure 2:**
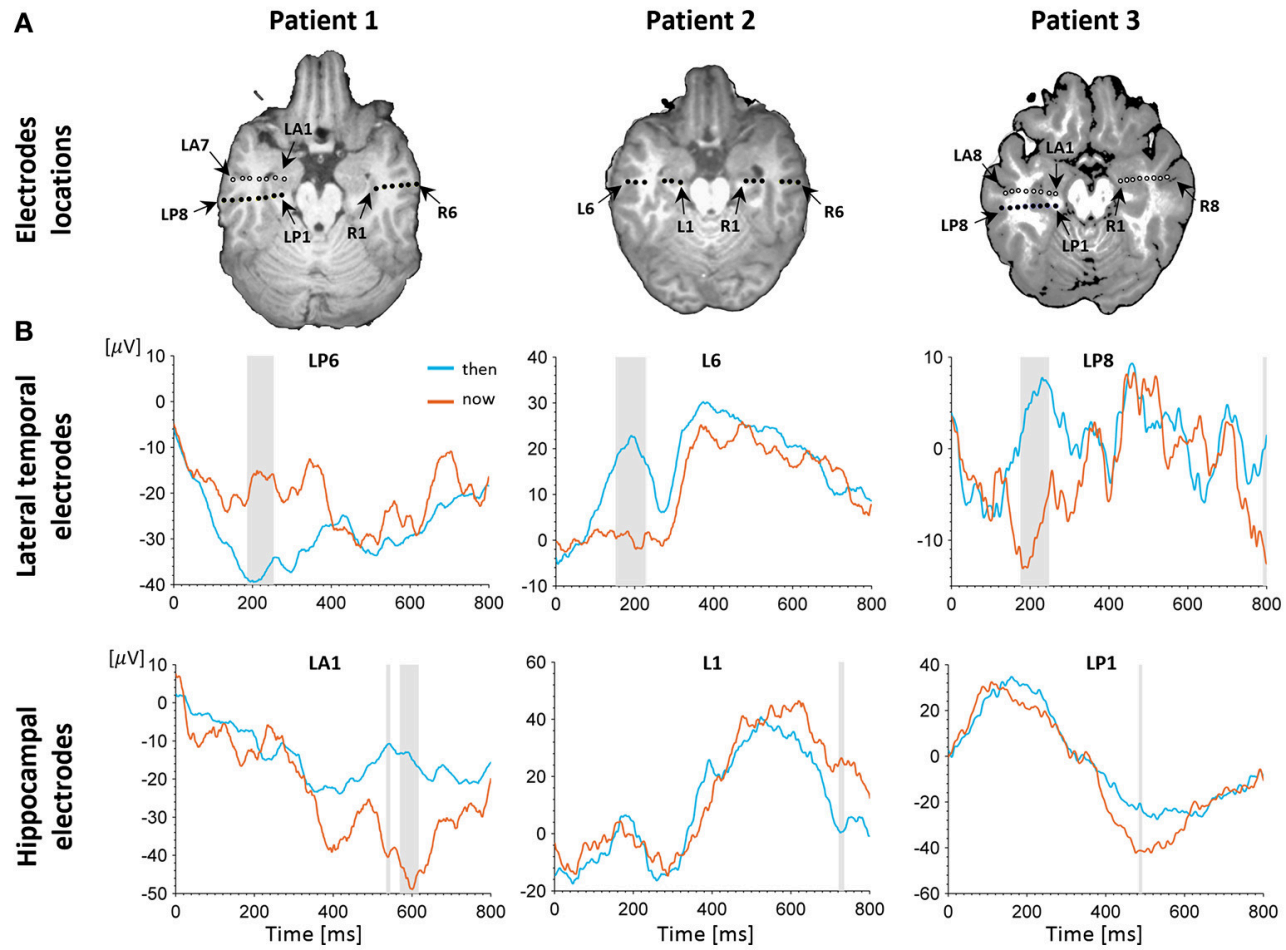
Electrophysiological results. **(A)** Depth electrodes locations in the hippocampus and lateral temporal cortex (LTC), shown for each patient on a co-registration of post-operative CT scan and pre-operative MRI (white circles depict electrodes projected on this slice for visualization purposes; for more precise localization of these electrodes see Figure S2. Exact neuroanatomical position of each electrode as verified by two certified neuro-radiologists is available in Table S1. **(B)** Intracranial evoked potentials (iEPs) recorded at representative electrodes in the left LTC (top) and left hippocampus (bottom) during MTT. LTC electrodes show high early task modulation, whereas electrodes in the hippocampus show high late task modulation. Shaded areas show time points of significant differences between conditions in a two-tailed independent samples *t*-test (*p* < 0.05, uncorrected).

Patients were implanted with depth electrodes penetrating the temporal lobe from the neocortex to the MTL bilaterally according to strict clinical criteria. In total, we have analyzed 57 electrodes implanted in all three patients (Figure 2A).

### EEG acquisition and analysis

Continuous intracranial EEG was acquired with a Deltamed® system [1,024 Hz (patients 1,2) or 512 Hz (patient 3) digitization]. Depth electrodes had a center-to-center distance of 1 cm (Ad-Tech, Racine, WI). Electrode location was determined by three dimensional MRI of the brain as well as CT scan with the implanted electrodes (Blanke et al., 1999, 2005). Preprocessing and analyses were conducted using Cartool software (Brunet et al., 2011; https://sites.google.com/site/cartoolcommunity/), Brainstorm toolbox (Tadel et al., 2011; http://neuroimage.usc.edu/brainstorm), FieldTrip toolbox (Oostenveld et al., 2011; http://www.ru.nl/neuroimaging/fieldtrip), and Matlab® (Mathworks, inc.). Epochs of EEG from 100 ms before to 800 ms after stimulus onset were bandpass filtered (1–120 Hz), and averaged for each of the stimulus conditions to calculate the intracranial evoked potential (iEPs). In the MTT task, the past and future conditions were collapsed into one condition (“then”), allowing a simpler 2 × 2 design (now/then × before/after) in accordance with previous studies showing similar response to past and future events (e.g., Arzy et al., 2008, 2009a; Anelli et al., 2016; Gauthier and van Wassenhove, 2016; for review see Schacter et al., 2012). Data were inspected visually to reject epochs with epileptic discharges as well as epochs with other types of transient noise.

### Electrode selection

We aimed to differentiate between lateral cortical and hippocampal activations in response to the MTT and the spatial tasks. To this end, we identified hippocampal and LTC electrodes according to their apparent location on a post-implantation CT, co-registered with the pre-implantation MRI images. Exact neuroanatomical position of each electrode was verified by two certified neuro-radiologists using a neuroanatomical atlas (Harnsberger et al., 2006). Electrodes that showed clearly defective iEPs were excluded from the analyses.

### Electrodes classification

Following our previous findings using EEG (Arzy et al., 2008), we defined two time periods of interest: an early period ranging from 100 to 400 ms post stimulus onset that encompassed the initial peak responses at the LTC, and a late period ranging from 400 to 800 ms post stimulus onset that captured a second peak response in the hippocampus (Figure 2B; Staresina et al., 2012). To differentiate between LTC and hippocampal electrodes we defined early and late modulation features for each electrode and task, as follows (Figure 4): For each condition and period, the raw modulation was defined as the absolute value of the sum of differences between iEPs deflections in the two conditions (the signed area between the two iEPs deflections). Subsequently, the modulation was normalized by the area under the curve of the “now” (or “here”) condition in the same period. Accordingly, the early modulation of electrode *i* in the time-task is given by:

(1) $$\begin{array}{r} Early\;modulation=\frac{\left|\int_{100ms}^{400ms}S_{then}^{i}\left(t\right)-S_{now}^{i}\left(t\right)dt\right|}{\left|\int_{100ms}^{400ms}S_{now}^{i}\left(t\right)dt\right|} \end{array}$$

Where $S_{now}^{i}\left(t\right)$ and $S_{then}^{i}\left(t\right)$ are the mean iEPs recorded in electrode *i* in the “now” and “then” conditions, respectively. Likewise, the late modulation is defined with integration limits of 400–800 ms.

Each electrode's position in the two dimensional feature space was thus determined by its early and late task modulations (Figure 4D). When lateral temporal and hippocampal electrodes seemed separable in this representation, we tested for significance of this separation using Support Vector Machine with a linear kernel (SVM; Cortes and Vapnik, 1995). Linear SVM is a supervised learning algorithm that performs linear classification of the data by constructing the optimal hyperplane with largest margin for separating data into two groups. To avoid domination of small numeric results by greater ones we scaled the data by Z-score procedure for each of the two features (Chang and Lin, 2011).

SVM uses a penalty parameter *C* > 0 that determines the tradeoff between margin maximization and training error minimization. An optimal value for this parameter had to be determined. Ten different *C*-values equally spaced on a log-scale in the range of [10^−3^,10^3^] were tested, each yielding a cross-validation classification accuracy using the N-fold cross-validation procedure (Chang and Lin, 2011). The *C*-value yielding the highest cross-validation accuracy was subsequently used for training the classifier and for statistical tests.

To statistically validate our classification results, we used a non-parametric permutation test (Ojala and Garriga, 2010). The null hypothesis of this test is that the dataset labels (LTC or hippocampal) are independent of the features (early and late modulations). We re-trained the classifier on all possible permutations of the dataset labels, and calculated the N-fold cross-validation accuracy for each permutation. This allowed the derived classification accuracy to be assigned a *p*-value. In case the dataset labels and features are independent in the original data, one can expect to obtain high *p*-values (Ojala and Garriga, 2010).

### iEP-amplitude analysis

We examined whether iEPs significantly differed between conditions (“now”/“then” and “here”/“there”). To this aim, statistical analysis (*t*-tests, two tailed, *p* < 0.05, uncorrected) was used on the amplitude of the single unaveraged epochs over trials, comparing the different experimental conditions in each time-frame, and searching for significant differences. Since iEP values at adjacent time-frames are highly dependent, one cannot use conventional methods of correction for the multiple comparisons. We therefore used a cluster-based nonparametric randomization test (Maris and Oostenveld, 2007). In short, clusters were defined as continuous time-frames in which the t-statistic exceeded a given threshold (corresponding to *p* < 0.05). A cluster-level test statistic was defined as the sum of all t-statistics in the cluster, and the type-I error rate was controlled by evaluating the cluster-level test statistic under the randomization null distribution of the maximum cluster-level test statistic, using 1,000 random permutations between the two conditions and *p* < 0.05.

## Results

A behavioral self-projection effect was found in two out of the three patients, with longer reaction times for the “past” and “future” conditions compared with the “now” condition (*p* < 0.05 for all tests), comparable to previous studies using the same paradigm in larger number of subjects (e.g., Arzy et al., 2008, 2009a). To distinguish between LTC and hippocampal involvement we used data from all patients and analyzed 12 electrodes in the left hemisphere (six in the LTC and six in the hippocampus) and eight electrodes in the right hemisphere (three in the LTC and five in the hippocampus; Figure 2A, Figure S2). Analysis of iEPs in the left hemisphere in the MTT task showed a significant early task modulation in the time window of ~100–300 ms (*p* < 0.05 uncorrected) in five out of six LTC electrodes (Figure 2B, upper row; Figure S3). A late task modulation was found in the time window of ~400–600 ms in all hippocampal electrodes (Figure 2B, lower row; Figure S3). Such consistent effects were not found in the right hemisphere (Figure S4), nor in the spatial task in either hemisphere (Figures S6, S7).

**Figure 3:**
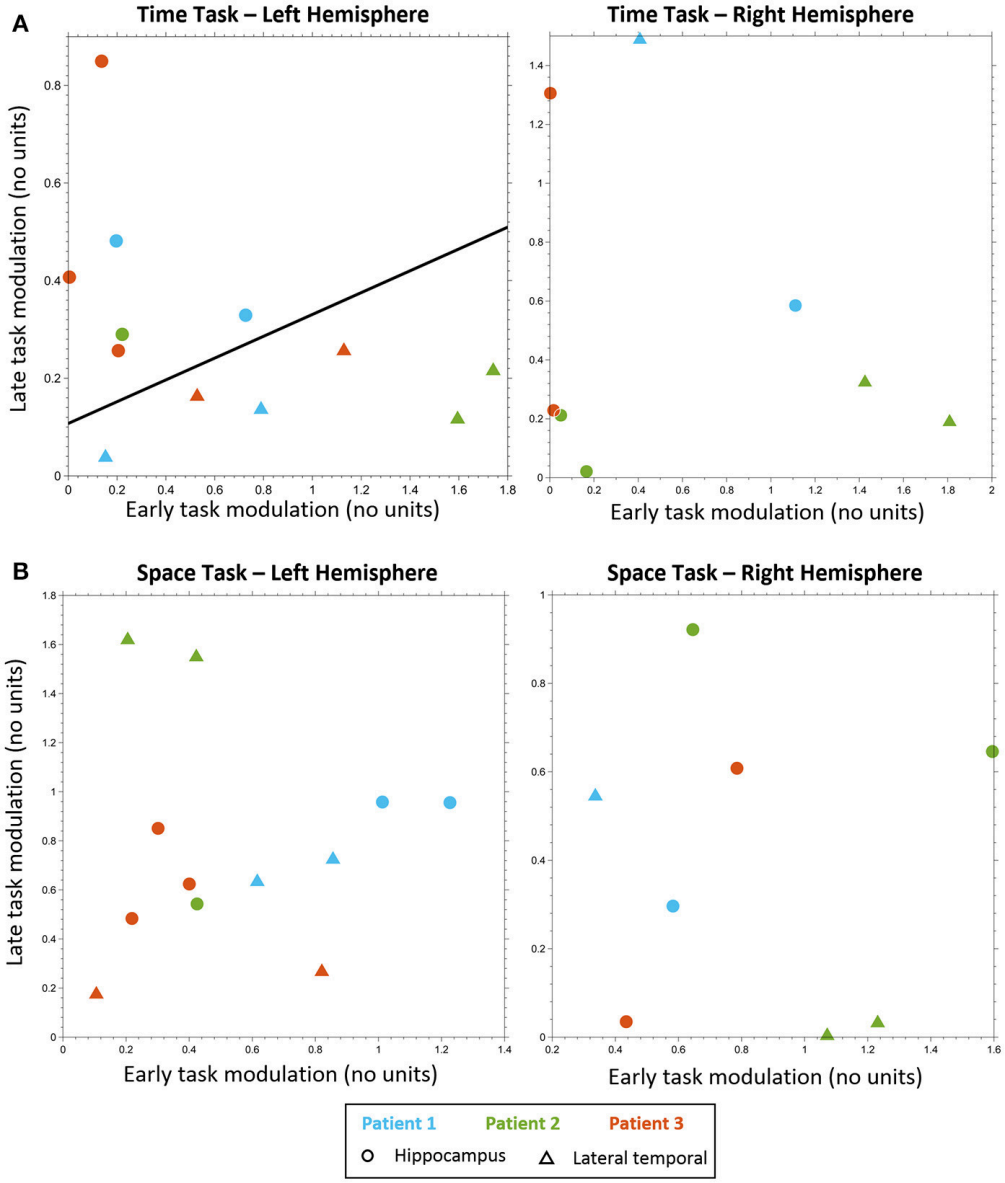
Electrodes classification. Electrodes classification using linear SVM, based on early task modulation value (X-axis) and late task modulation value (Y-axis). **(A)** MTT task: left hippocampal electrodes (circles) are clearly separable from left lateral temporal cortex (LTC) electrodes (triangles) on the plane of early and late task modulations (see Figure S3 and Table S2). A separating line is shown, as obtained from SVM classification of all electrodes (left, *p* < 0.005). No such separation was found for electrodes in the right hemisphere (right, see Figure S4). **(B)** Spatial task: no separation between lateral temporal and hippocampal electrodes was found neither in the left hemisphere (left, see Figure S6) nor in the right (right, see Figure S7).

Classification analysis based on the early and late task modulations (Figure 3) yielded a significant separation between LTC and hippocampal electrodes in the MTT task in the left hemisphere (cross-validation accuracy 100%, *p* = 0.004; Figure 3A). Five out of six electrodes which showed late hippocampal modulation were located in the hippocampal formation (HF) and one in the parahippocampal gyrus. No significant separation was found in the right hemisphere (cross-validation accuracy 75%, *p* = 0.304; Figure 3B), nor in the spatial task either for the left or right hemispheres (cross-validation accuracy 33.33, 62.5%; *p* = 0.847, 0.982, respectively; Figures 3C,D). No significant difference between conditions was found in the MTT task nor in the spatial task using the cluster-based nonparametric randomization test.

**Figure 4:**
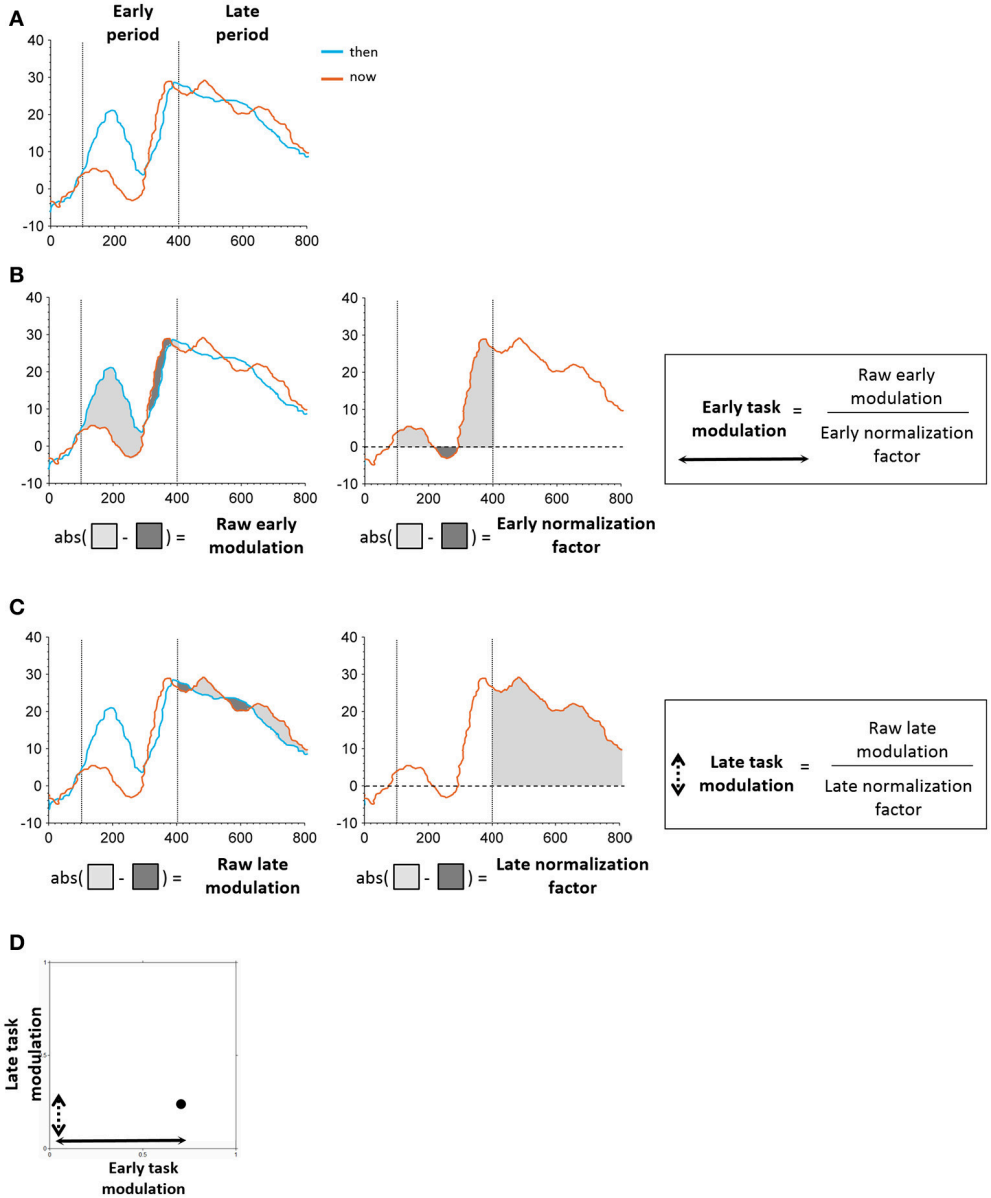
Schematic illustration of task modulation extraction. **(A)** Early and late periods identified in the time windows of 100–400 and 400–800 ms post stimulus onset, respectively. **(B)** Extraction of early modulation value. The raw modulation was defined as the absolute value of the sum of differences between iEPs in the two conditions (“then”-“now,” “there”-“here”; left). The normalization factor was defined as the area under the curve of the “now”/“here” condition in the respective period (middle). The raw modulation was subsequently normalized by the normalization factor of the respective period, resulting in the final task modulation value (right). **(C)** Extraction of early modulation value. Same procedure as applied for the early task modulation was used here. **(D)** Each electrode's position in the two-dimensional feature space was determined by its early and late task modulation values.

## Discussion

The present study used the high temporal and spatial resolution of intracranial recordings and employed a classification analysis in order to distinguish between LTC and hippocampal involvement in self-projection in time, a key component in MTT. Our iEP data revealed that LTC and hippocampal contributions to self-projection in time display distinct temporal dynamics. Classification analysis of electrodes in the left hemisphere showed a clear temporal dissociation between LTC electrodes that exhibited an early self-projection component (~100–300 ms), and hippocampal electrodes that exhibited a late component (~400–600 ms). No such effect was found either in the right hemisphere or in a control task of self-projection in space.

Our results suggest the involvement of both LTC and the hippocampus in MTT. Several neuroimaging studies involving MTT-related tasks revealed increased activation in both the medial temporal lobe and the LTC (Addis et al., 2007, 2009a, 2011; Buckner and Carroll, 2007; Schacter and Addis, 2007; Botzung et al., 2008; Arzy et al., 2009a; Spreng et al., 2009; Spreng and Grady, 2010; Schacter et al., 2012; Benoit and Schacter, 2015). The high spatial and temporal resolution of iEPs enabled us to temporally dissociate the contributions of these two regions during MTT. We believe these results could not be explained by mere temporal delay in the processing of the same information at the circuit level, since other sEEG studies have identified hippocampal responses within the first few 100 ms of stimulus/task onset (Axmacher et al., 2007, 2010; Olsen et al., 2012), while here hippocampal activity was found significantly later (~400–600 ms). Therefore, these results suggest a division of labor in the temporal lobe: Early processing of self-projection takes place in the LTC, to establish one's self-location on the mental time line (the first step in the MTT task). Subsequently, hippocampal activity possibly reflects the required computations for orienting oneself with respect to the presented events (the second step in the MTT task). These results are in line with patient data revealing preservation of self-projection effects despite hippocampal lesions (Arzy et al., 2009b). This latter implication of the hippocampus in MTT may be related to its role in determining the temporal order of events, in accordance with the “relational memory theory” (Eichenbaum and Cohen, 2014). According to this theory, the hippocampus serves as a general relational processing mechanism, involving, among other representational schemes, the representation of episodes as the flow of events across time. The hippocampus may be similarly involved in the task used here, in determining the temporal relations of the events to one's imagined self-location in time. This is also in line with previous clinical and neuroimaging studies that found hippocampal activity in tasks involving general relational processing (Giovanello et al., 2004; Preston et al., 2004; Prince et al., 2005; Konishi et al., 2006), and specifically in the context of the temporal order of events (Reber and Squire, 1998; Hopkins et al., 2004; Lehn et al., 2009; Paz et al., 2010; Davachi and DuBrow, 2015; Rubin et al., 2015; Jenkins and Ranganath, 2016). Impaired ability to explicitly remember the sequential order of events was also found in studies in amnestic patients with hippocampal damage (Reber and Squire, 1998; Hopkins et al., 2004) as well as lesion studies in nonhuman animals (DeCoteau and Kesner, 2000; Fortin et al., 2002; Kesner et al., 2002).

Most hippocampal electrodes that showed late hippocampal modulation were located in the hippocampal formation (HF). The HF has been shown to be involved in MTT and autonoetic consciousness in a unique model of patient population with a specific lesion in the CA1 part of the HF (Bartsch et al., 2011). In a more precise manner, the HF also contains the recently discovered time-cells. Accumulating experimental evidence, mostly in rodents but also in humans, suggest that the hippocampus plays a central role in the temporal organization of memories (Devito and Eichenbaum, 2011; for review see Eichenbaum, 2013). Notably, these cells share similar properties with place-cells, which encode one's location in the environment (Kraus et al., 2015). Likewise, a time-space similarity was recently found in the distributed manner in which episodic or atemporal spatial memories are represented along the hippocampal axis, based on their temporal or spatial scale (Collin et al., 2015). However, such a similarity between the hippocampal responses to the MTT and spatial tasks was not evident in our results. A potential reason for that is that the spatial task here is not equivalent to the MTT task. Future studies may better address this point by designing more comparable temporal and spatial tasks (e.g., Gauthier and van Wassenhove, 2016). Another possibility is that higher-order functions as examined here are not directly related to time- and place-cells, which could be responsible for encoding much shorter distances and time-scales.

Previous studies established the LTC as part of the MTT network, supporting both episodic memory and episodic future thinking (Svoboda et al., 2006; Hassabis et al., 2007a; Addis et al., 2009a; Spreng et al., 2009; Markowitsch and Staniloiu, 2011; Benoit and Schacter, 2015). Nevertheless, its exact role in the different processes comprising MTT is not completely clear. Much evidence has accumulated relating LTC activity to retrieval of semantic memory, by means of neuroimaging studies of various memory tasks in healthy subjects (Martin and Chao, 2001; McClelland and Rogers, 2003; Konishi et al., 2006), as well as studies in patients who suffered damage to the LTC (Hodges et al., 1992; Gilboa et al., 2005; Addis et al., 2009b). Retrieval of semantic knowledge has been suggested to subserve both recollection and future thinking, and thus support MTT (Tulving, 2002; Levine, 2004; Schacter et al., 2012). Recruitment of LTC was found in tasks involving decision making with respect to personal events (Andrews-Hanna et al., 2010), self-projection in time (St Jacques et al., 2011), construction and elaboration of past and future events (Addis et al., 2007), and orientation with respect to different events in time (Peer et al., 2015). The early iEP modulation we found in LTC further established the notion that the LTC supports MTT not only via retrieval of semantic information, but also through direct involvement in the act of self-projection in time.

Significant separation of the LTC and the hippocampus based on their temporal pattern of activity was found in our study only in the left hemisphere. Lateralization in the hippocampi has been known for a long time, but less so is the lateralization in the LTC. Our results are concordant with previous studies that found predominant left lateralization in various tasks involving autobiographic memory and orientation in time (Maguire, 2001; Levine, 2004; Svoboda et al., 2006; Arzy et al., 2008; Spreng et al., 2009; Peer et al., 2015), though some other studies have suggested right predominance (Fink et al., 1996; Gilboa et al., 2005; Arzy et al., 2009a). It should be noted that while our results suggest left lateralization, the lack of effect in the right hemisphere should be interpreted with caution. Due to the small number of electrodes that met inclusion criteria in the right hemisphere (8 overall, where no LTC electrodes were included for subject 3), classification in this hemisphere is of limited value. In an additional analysis in which the number of electrodes in the left hemisphere was reduced to match that of the right hemisphere, the power of the test was indeed reduced, as expected (see Box 1 and Figure S8). This is indeed a main limitation of this study, which includes a relatively small number of patients. However, this sample size is comparable to several other studies that include intracranial recording in human hippocampus (Vanni-Mercier et al., 2009; Staresina et al., 2012; Kurczek et al., 2015). Such small samples are customary due to the rare opportunity to record intracranial artifact-free high-quality electrophysiological data in response to high-cognitive tasks such as MTT and self-projection, which is not applicable even in primates. Notably, most patients with temporal electrodes suffer from hippocampal sclerosis and frequent electrical discharges, which contaminate the data. Such patients were not included in our study, making the study sample of high quality, though small. Moreover, our results were consistent across all subjects. Subjects were nevertheless epileptic patients in whom interictal epileptic activity may influence results. To avoid such a disturbance we applied several methods: First, in two of our patients epileptic foci were identified elsewhere and in one aberrant epileptic activity was absent during recording as well as 2 days later. The data was also inspected visually to exclude any epileptic artifacts. Stimulus-locked iEPs were clear and similar among patients. Most late modulations were found in the HF. However, more electrodes in other hippocampal locations may show responses as well. This was nevertheless impossible to test in our study, due to strict clinical considerations regarding electrodes implantation. It should thus be noted that the HF effect found here does not exclude a parallel parahippocampal effect.

Box 1 **The effect of reducing the number of electrodes used in the classification analysis.**

In our study we found significant separation of the LTC and the hippocampus based on their temporal pattern of activity only in the left hemisphere during the time task. Although these results seem to support left lateralization, the lack of clear separation in the right hemisphere should be interpreted with caution. Due to the small number of electrodes that met inclusion criteria in the right hemisphere (8 overall, where no LTC electrodes were included for subject 3, compared with 12 overall in the left hemisphere), classification in this hemisphere is of limited value. In other words, it is possible that the power of the statistical method used in this study is too low to reveal an effect in the right hemisphere, even if it exists. In principle, one could estimate the number of electrodes required to obtain a certain power level of the test, yet general procedures for planning sample size are yet to be developed in the case of classification based tests (Maxwell et al., 2008).

To assess the effect of the small number of electrodes in the right hemisphere, we conducted an additional analysis in which the number of electrodes in the left hemisphere was reduced to match that of the right hemisphere. The same classification analysis was done for all 120 possible subsets of electrodes in the left hemisphere which include exactly 5 hippocampal electrodes and 3 lateral temporal electrodes, as in the right hemisphere. For each subset we calculated the cross-validation accuracy and its *p*-value (see Materials and Methods). Figure S8 shows the distribution of resulting accuracy values and their corresponding *p*-values. Although high accuracy values (>75%) were found in a large number of electrodes subsets (84/120), these findings were significant (*p* < 0.05) for only a small fraction of the subsets (33/120). These results suggest that the lack of significant temporal separation in the right hemisphere could be the result of reduced power of the statistical analysis due to the small number of electrodes in this hemisphere.

As noted earlier, the spatial task is not equivalent to the time task. However, in both tasks patients had to imagine themselves in a different self-location—in time or in space. The absence of a significant early component for space in the LTC is also supported by fMRI and EEG studies using the same space task, which did not show such an activation (Arzy et al., 2006; Ionta et al., 2013). The late hippocampal modulation which relates stimuli to the projected self may be absent due to the nature of the spatial task used. Further study of a comparable spatial task involving relational organization of self and landmarks in space could shed light on the role of the hippocampus in non-temporal relational organization (Gauthier and van Wassenhove, 2016). We therefore refer in this study mostly to results found in the MTT task and mention spatial task results with caution.

Our small number of patients did not allow for reliable statistical testing using conventional approaches. Specifically in intracranial studies, it is difficult to delineate consistent iEPs across individuals, in part due to varying relative positions of the electrodes across different subjects. For example, such variability leads to “polarity reversal” (Halgren et al., 1982): When recording iEPs from local generators, the polarity of the resulting iEP reverses as one records from two opposite sides of this generator (Figure S5). We therefore suggest that classification, done at a low dimensional feature space that summarizes the iEPs recorded at each electrode, is a more suitable statistical method in such cases, and may serve as a useful tool in analyses of other neuroscientific data as well (Box 2; see also Arzy et al., 2014). While classification reliably distinguishes between predefined classes, the applied predefinition inevitably influences the results. Classification here was nevertheless based on previous results using fMRI and EEG, enabling a precise predefinition of classes with respect to neuroanatomical localization and appropriate time windows, respectively.

Box 2 **Statistical learning and classification in the analysis of intracranial data.**

Intracranial electrophysiological recording in awake human patients is the most accurate existing method in the cognitive neurosciences. Unlike non-invasive methods—such as functional MRI, MEG or EEG—it enables direct recording of neural activity in exceptionally high spatial and temporal resolutions, as well as a high signal to noise ratio (SNR; Lachaux et al., 2003; Ball et al., 2009). It is therefore the only manner by which electrophysiological correlates of high cognitive functions may be recorded invasively, since such functions cannot be controlled in non-human animals, including primates. However, statistical group analysis—a common approach in the abovementioned modalities—is difficult to employ in iEPs. This is due to the strict clinical considerations regarding location of electrodes implantation and experimental settings, which ultimately lead to significant variability among individual patients. Therefore, whereas other neuroimaging methods are used to identify group effects across many subjects, in iEPs experiments, where only a handful of patients are usually recruited, analysis is effectuated in the individual subject level (Kramer et al., 2011; Peer et al., 2015). While the high quality of the data could enable the detection of significant effects on the level of individual subjects, it is not free of limitations. Statistics is done over trials, which do not necessarily reflect the cognitive effect; the number of repetitions affects both subjects' performance and statistical power; correction for multiple comparisons is dependent on the number of electrodes, which, in turn, are inserted according to clinical considerations and differ between patients. Needless to mention, even classical group effects are prone to invalid statistical inferences due to low statistical power, improper circular analysis, or other biases that tend to increase false-positive rates (Kriegeskorte et al., 2009; Simmons et al., 2011; Button et al., 2013).

A statistical method that may overcome these caveats, and therefore is appropriate for the analysis of iEP data, is *statistical learning*, and specifically *classification* (Arzy et al., 2014; Shalev-Shwartz and Ben-David, 2014). Here we use a distribution-free framework, aiming to identify a classification rule by which a new observation can be classified as belonging to one class or another. The classification process and resulting predictions are based on a set of features inherent to the data (e.g., in iEPs features may be comprised of amplitude, latency or power spectra, or as in our case: late and early task modulations). Each observation, or *instance*, is represented as a “vector of features” in the features space. Instances are further *labeled* as belonging to one of two or more predefined *classes* (e.g., in iEPs classes may consist of anatomical electrode location such as hippocampal vs. LTC, different frequency bands, or experimental conditions). In the framework of *supervised learning*, a finite set of labeled instances is defined as the *training data*. Subsequently, the procedure produces a *predictor*, or *classifier*, which can be used to predict the label of new instances, by separating the instances to different classes according to a certain *classification rule* (e.g., distance to its nearest neighbors or linear separation). The *accuracy* of a classifier is the probability that it will predict the correct label on a randomly generated set of instances and can be estimated on a given instance set using the N-fold cross-validation procedure (also termed “leave-one-out cross-validation”; Chang and Lin, 2011). In this procedure, classification is learned using N-1 instances, and then used to predict the label of the remaining instance. The process is repeated N times, and the fraction of instances classified correctly is used as the estimated classifications accuracy. In addition, one may estimate the statistical significance of classification accuracy by using methods such as non-parametric permutation tests on the dataset labels. Overall, such a statistical learning approach may therefore fit well iEPs analysis, as long as the research question may be reformulated as a classification problem into two (or several) predefined *classes*.

To conclude, in the present study we found that both the LTC and the hippocampus are involved in MTT; however, while the first is involved early in the process, as subjects “project” themselves in time, the latter is only involved later, when subjects relate the different events to the “projected” self. This division of labor may contribute to the reconciliation of the major debate regarding the role of the hippocampus in MTT.

## Author contributions

SA and OB: Designed the study; SA, LS, and MS: Performed the study; RS, MN, and SA: Analyzed the data; RE: Analyzed the neuroanatomical structures; RS and SA: Wrote the manuscript.

### Conflict of interest statement

The authors declare that the research was conducted in the absence of any commercial or financial relationships that could be construed as a potential conflict of interest.

## Supplementary material

Figure S1 Own-body transformation task: Participants viewed a schematic human figure with one hand marked, facing either toward them or away from them. In the ‘here’ condition participants were asked to judge from their own self-location whether the marked hand was on the right or the left side of the computer screen. In the ‘there’ condition, participants were asked to “project” themselves to the position represented by the schematic human figure, and from this self-location to indicate whether the marked hand would be their right or left hand. Correct responses for each case are indicated below each figure.

Figure S2 Depth electrodes locations in the hippocampus and lateral temporal cortex (LTC), shown on individual patients' MRI scans.

Figure S3 Electrophysiological results for the time-task in the left hemisphere. Intracranial evoked potentials (iEPs) from all electrodes used in the classification analysis are presented. LTC electrodes (up) show high early task modulation, whereas electrodes in the hippocampus (bottom) show high late task modulation. Shaded areas show time points of significant differences between conditions in two-tailed independent samples t-test (*p* < 0.05, uncorrected).

Figure S4 Electrophysiological results for the time-task in the right hemisphere. iEPs recorded at electrodes in the right LTC and right hippocampus. No clear distinction in task modulation is apparent between LTC electrodes and electrodes in the hippocampus. Shaded areas show time points of significant differences between conditions in two-tailed independent samples *t*-test (*p* < 0.05, uncorrected).

Figure S5 Demonstration of iEPs polarity-reversal in the electrodes shown in Figure 1. Some iEPs in Figure 1 are of seemingly opposite polarity between Patients. This is the result of “polarity reversal” (Halgren et al., 1982). When recording iEPs from local generators, the polarity of the resulting iEP reverses as one records from two opposite sides of this generator. Observing such reversal in our data is expected since the exact relative position of electrodes differed between subjects. Note the iEPs similarity when plotting the reverse iEP (marked with an asterisk) in some of the electrodes.

Figure S6 Electrophysiological results for the space-task in the left hemisphere. iEPs from all electrodes used in the classification analysis are presented. No clear distinction in task modulation is apparent between LTC electrodes and electrodes in the hippocampus. Shaded areas show time points of significant differences between conditions in two-tailed independent samples *t*-test (*p* < 0.05, uncorrected).

Figure S7 Electrophysiological results for the space-task in the right hemisphere. iEPs recorded at electrodes in the right LTC and right hippocampus. No clear distinction in task modulation is apparent between LTC electrodes and electrodes in the hippocampus. Shaded areas show time points of significant differences between conditions in two-tailed independent samples *t*-test (*p* < 0.05, uncorrected).

Figure S8 The effect of reducing the number of electrodes used in the classification analysis. The distribution of cross-validation accuracy and corresponding *p*-values in the classification analysis of the MTT task, for subsets of 8 electrodes in the left hemispheres. Each subsets includes exactly 5 hippocampal electrodes and 3 lateral temporal electrodes, as in the right hemisphere. Although high accuracy values (>75%) were found in a large number of electrodes subsets (84/120), these findings were significant (*p* < 0.05) for only a small fraction of the subsets (33/120).

Table S1 Electrodes locations.

Table S2 Early and late modulation in time task, left hemisphere.
