# Supplementary figures and images for: Temporal Dissociation of Neocortical and Hippocampal Contributions to Mental Time Travel Using Intracranial Recordings in Humans
Source: Front Comput Neurosci. 2018 Feb 28;12:11. doi: 10.3389/fncom.2018.00011 (PMC5835533; doi:10.3389/fncom.2018.00011)

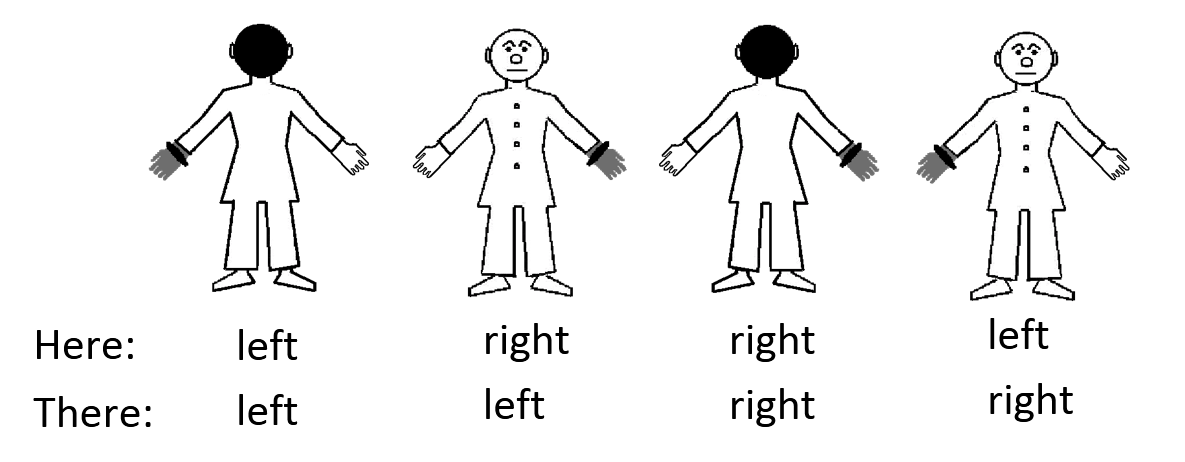

Supplement: Figure S1 — Own-body transformation task: Participants viewed a schematic human figure with one hand marked, facing either toward them or away from them. In the ‘here’ condition participants were asked to judge from their own self-location whether the marked hand was on the right or the left side of the computer screen. In the ‘there’ condition, participants were asked to “project” themselves to the position represented by the schematic human figure, and from this self-location to indicate whether the marked hand would be their right or left hand. Correct responses for each case are indicated below each figure. [file Image1.TIF]

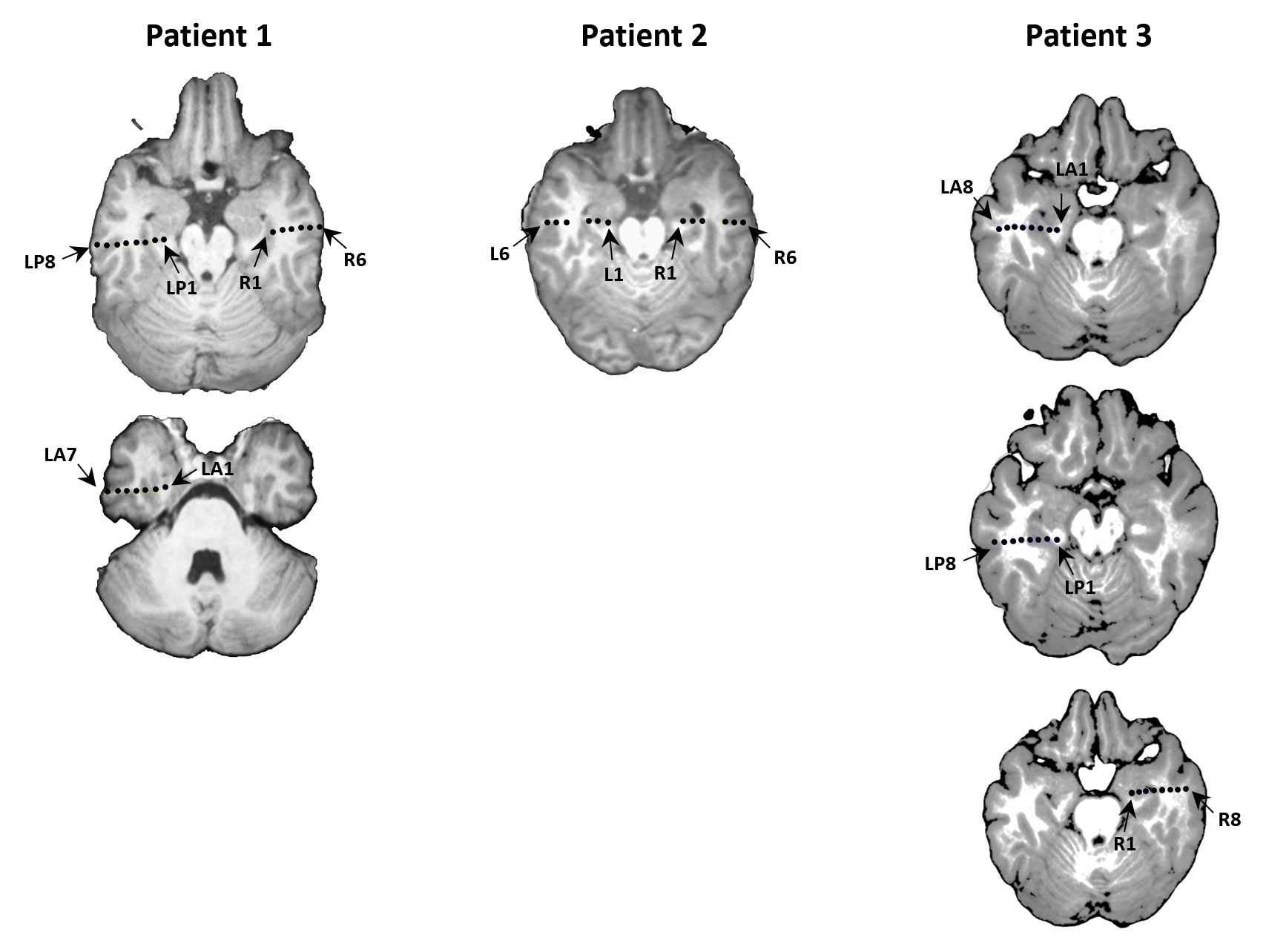

Supplement: Figure S2 — Depth electrodes locations in the hippocampus and lateral temporal cortex (LTC), shown on individual patients' MRI scans. [file Image2.TIF]

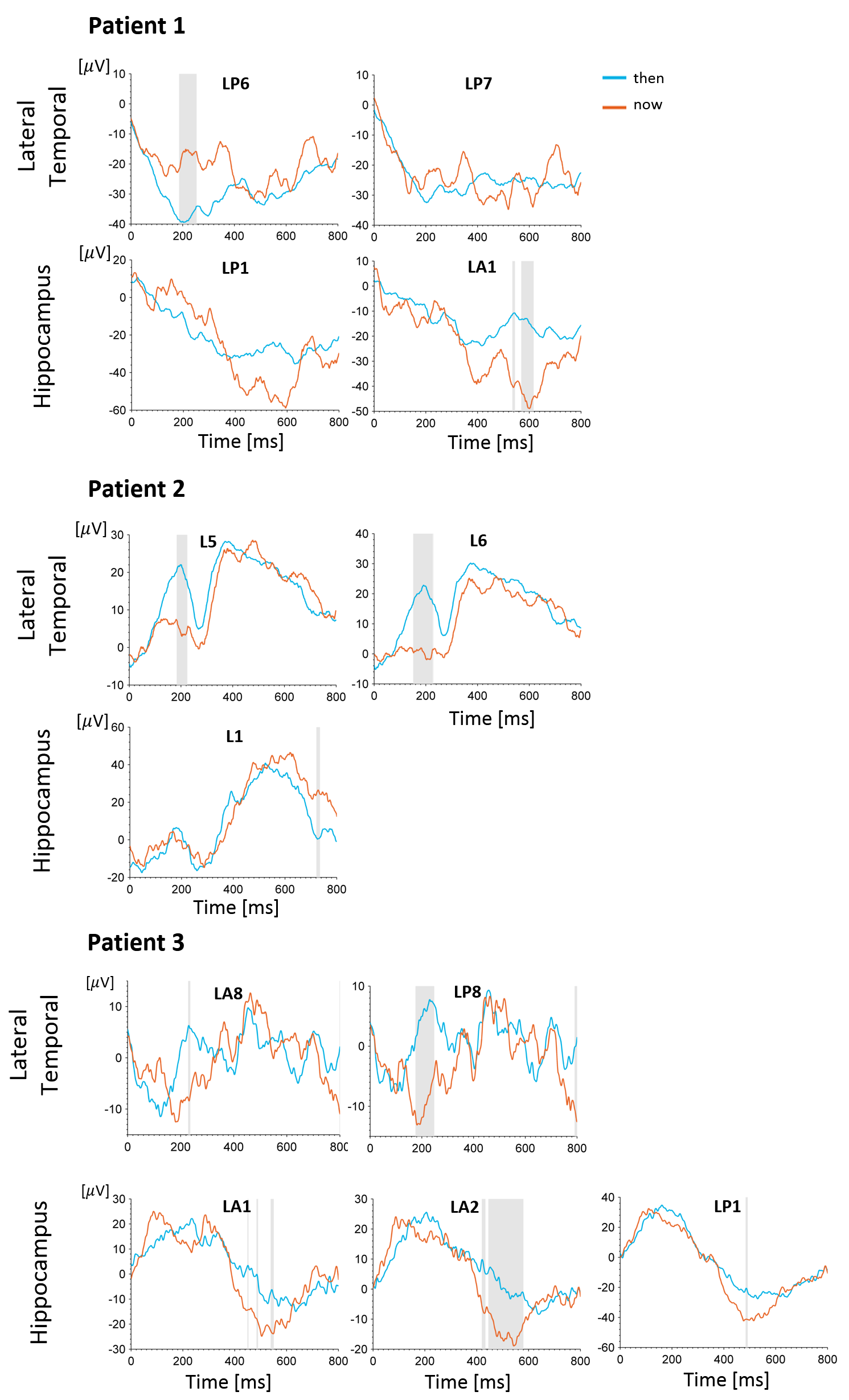

Supplement: Figure S3 — Electrophysiological results for the time-task in the left hemisphere. Intracranial evoked potentials (iEPs) from all electrodes used in the classification analysis are presented. LTC electrodes (up) show high early task modulation, whereas electrodes in the hippocampus (bottom) show high late task modulation. Shaded areas show time points of significant differences between conditions in two-tailed independent samples t-test (p < 0.05, uncorrected). [file Image3.TIF]

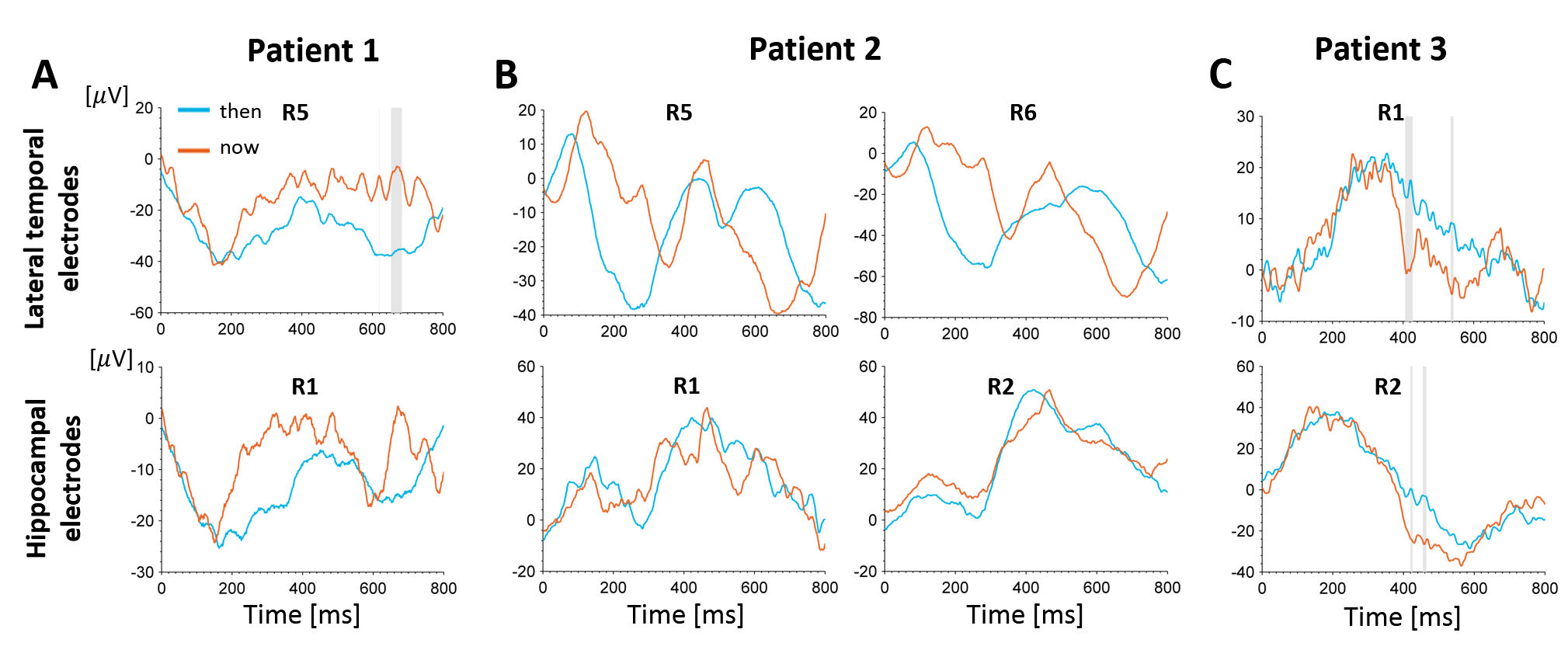

Supplement: Figure S4 — Electrophysiological results for the time-task in the right hemisphere. iEPs recorded at electrodes in the right LTC and right hippocampus. No clear distinction in task modulation is apparent between LTC electrodes and electrodes in the hippocampus. Shaded areas show time points of significant differences between conditions in two-tailed independent samples t-test (p < 0.05, uncorrected). [file Image4.TIF]

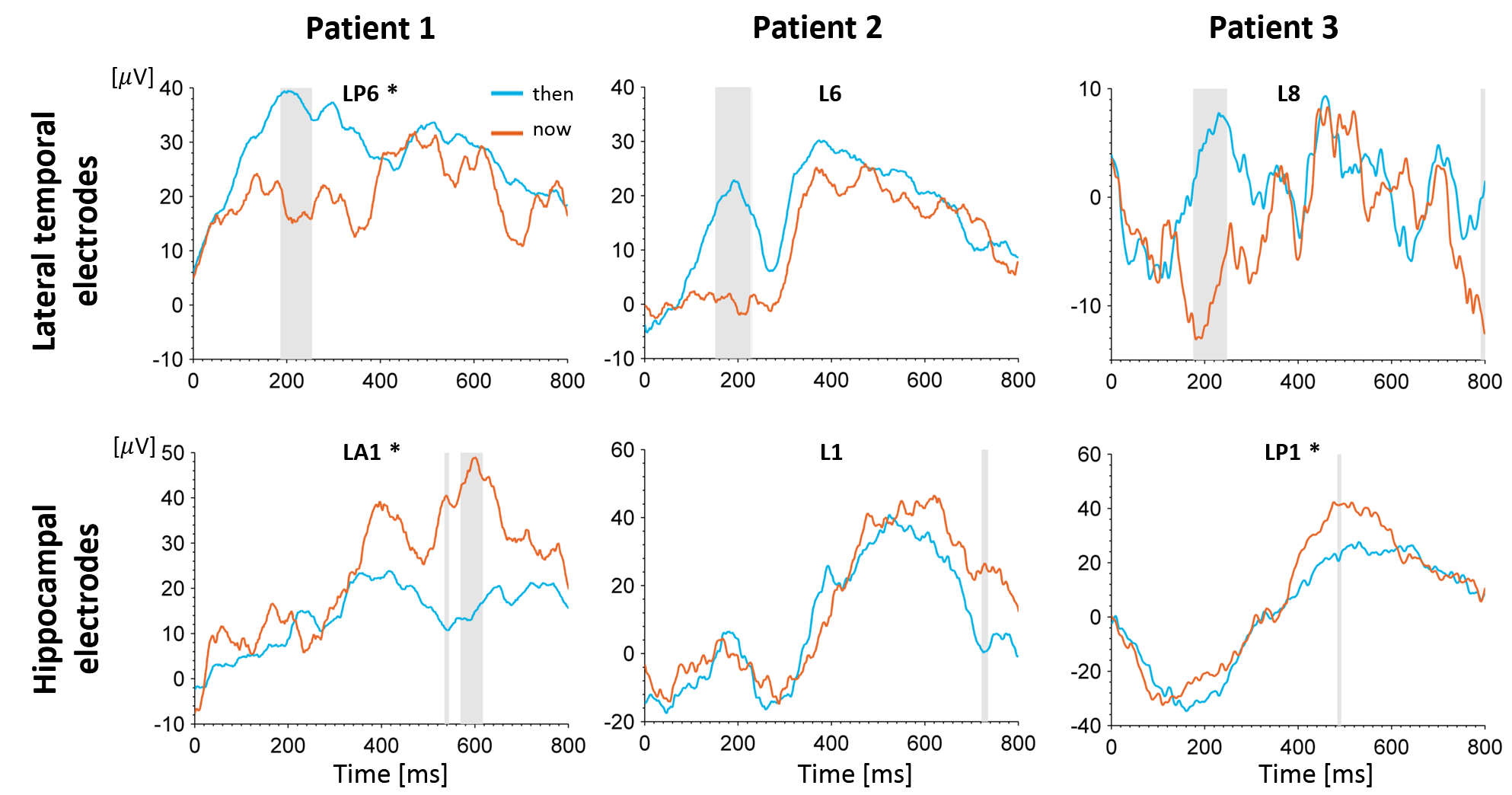

Supplement: Figure S5 — Demonstration of iEPs polarity-reversal in the electrodes shown in Figure 1. Some iEPs in Figure 1 are of seemingly opposite polarity between Patients. This is the result of “polarity reversal” (Halgren et al., 1982). When recording iEPs from local generators, the polarity of the resulting iEP reverses as one records from two opposite sides of this generator. Observing such reversal in our data is expected since the exact relative position of electrodes differed between subjects. Note the iEPs similarity when plotting the reverse iEP (marked with an asterisk) in some of the electrodes. [file Image5.TIF]

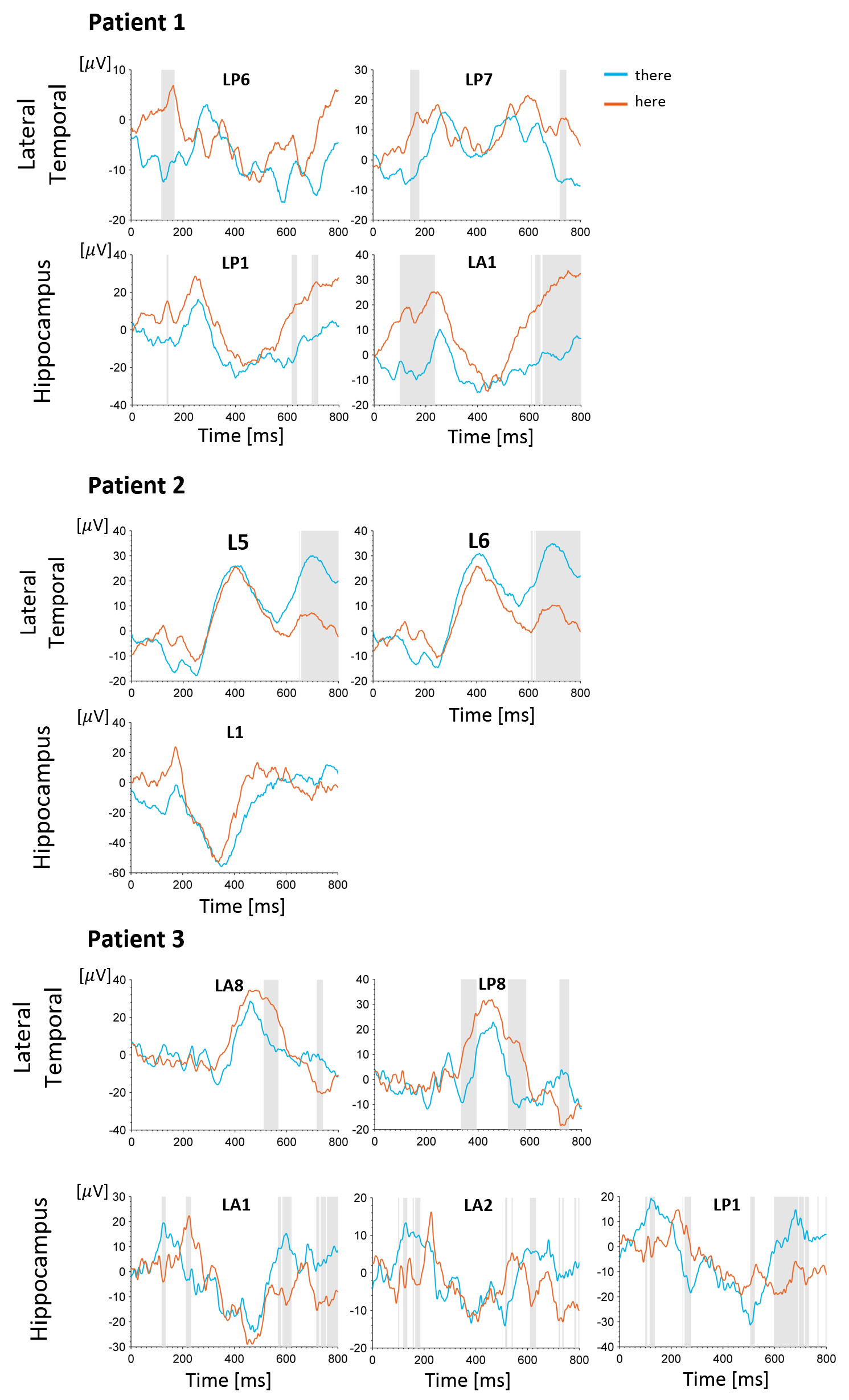

Supplement: Figure S6 — Electrophysiological results for the space-task in the left hemisphere. iEPs from all electrodes used in the classification analysis are presented. No clear distinction in task modulation is apparent between LTC electrodes and electrodes in the hippocampus. Shaded areas show time points of significant differences between conditions in two-tailed independent samples t-test (p < 0.05, uncorrected). [file Image6.TIF]

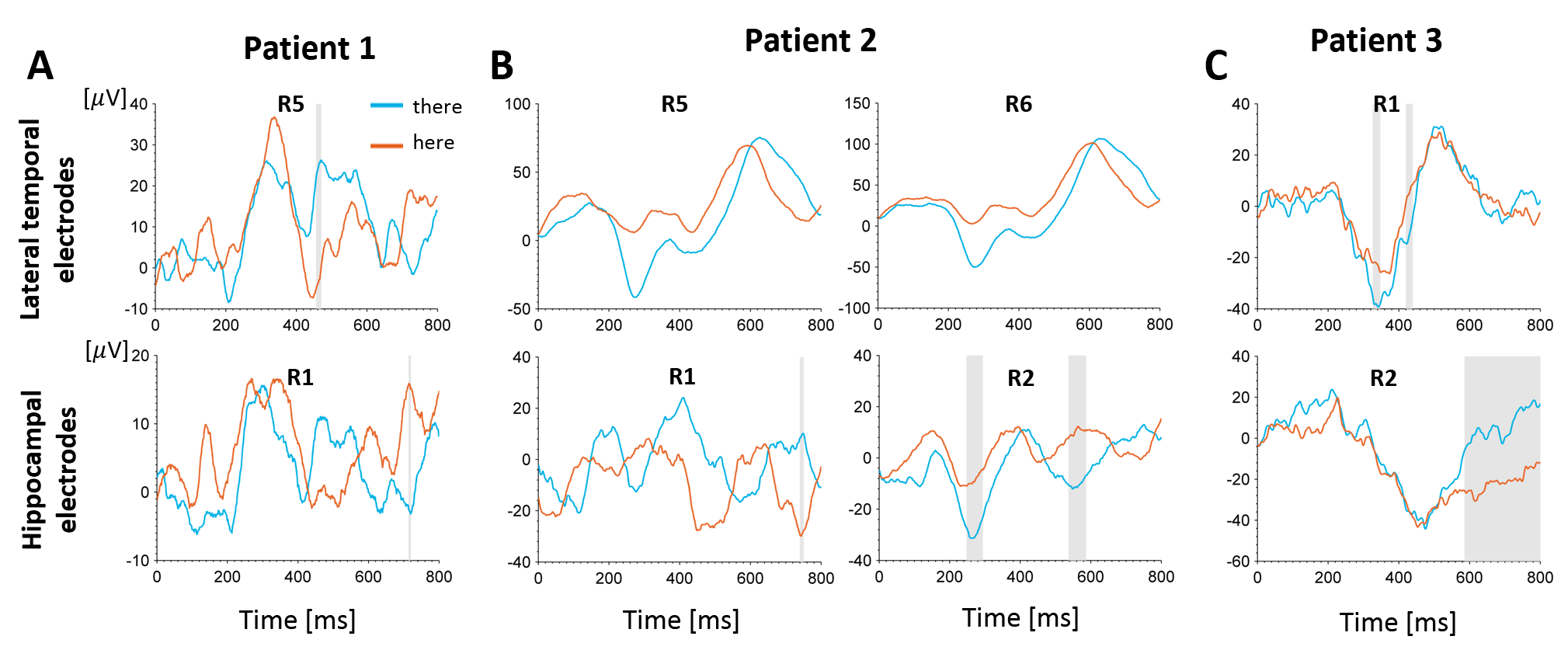

Supplement: Figure S7 — Electrophysiological results for the space-task in the right hemisphere. iEPs recorded at electrodes in the right LTC and right hippocampus. No clear distinction in task modulation is apparent between LTC electrodes and electrodes in the hippocampus. Shaded areas show time points of significant differences between conditions in two-tailed independent samples t-test (p < 0.05, uncorrected). [file Image7.TIF]

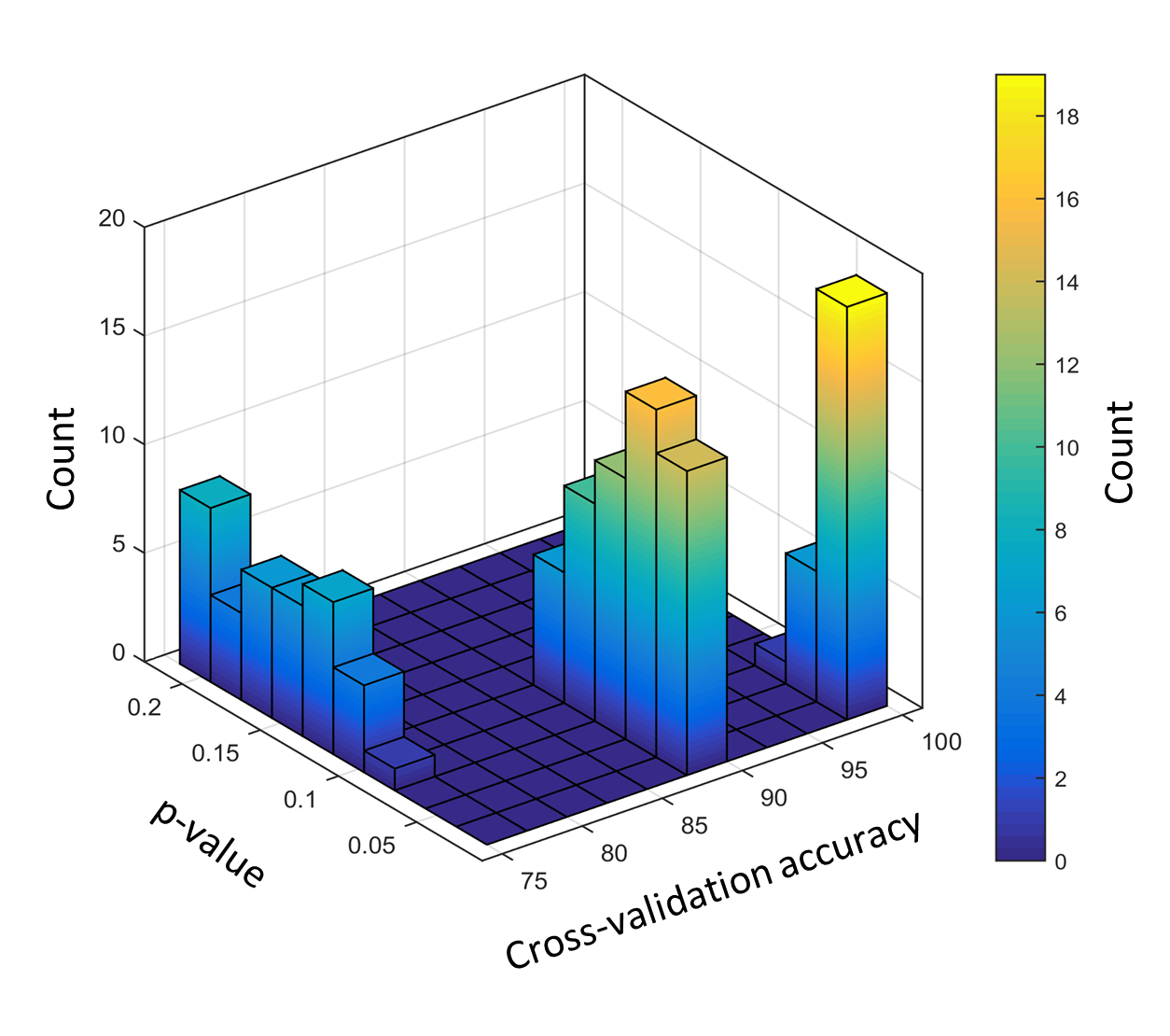

Supplement: Figure S8 — The effect of reducing the number of electrodes used in the classification analysis. The distribution of cross-validation accuracy and corresponding p-values in the classification analysis of the MTT task, for subsets of 8 electrodes in the left hemispheres. Each subsets includes exactly 5 hippocampal electrodes and 3 lateral temporal electrodes, as in the right hemisphere. Although high accuracy values (>75%) were found in a large number of electrodes subsets (84/120), these findings were significant (p < 0.05) for only a small fraction of the subsets (33/120). [file Image8.TIF]
