# Supplementary material for: Temporal Dissociation of Neocortical and Hippocampal Contributions to Mental Time Travel Using Intracranial Recordings in Humans
Source: Front Comput Neurosci. 2018 Feb 28;12:11. doi: 10.3389/fncom.2018.00011 (PMC5835533; doi:10.3389/fncom.2018.00011)
Supplement: Table S1 — Electrodes locations. [file Table1.DOCX]

**Electrodes locations**

| **Patient** | **Electrode** | **Location** |
| --- | --- | --- |
| **1** | LA1 | Hippocampal formation |
|  | LP1 | Subiculum |
|  | LP6 | Inferior temporal gyrus |
|  | LP7 | Inferior temporal gyrus |
|  | R1 | Hippocampal formation |
|  | R5 | Inferior temporal gyrus |
| **2** | L1 | Hippocampal formation |
|  | L5 | Middle temporal gyrus |
|  | L6 | Middle temporal gyrus |
|  | R5 | Middle temporal gyrus |
|  | R6 | Middle temporal gyrus |
| **3** | LA1 | Hippocampal formation |
|  | LA2 | Hippocampal formation |
|  | LP1 | Parahippocampal gyrus |
|  | LA8 | Between middle and inferior temporal gyri |
|  | LP8 | Between middle and inferior temporal gyri |

Location of the electrodes included in the classification analysis. L = left; R = right; A = anterior; P = posterior.
