# Supplementary material for: Temporal Dissociation of Neocortical and Hippocampal Contributions to Mental Time Travel Using Intracranial Recordings in Humans
Source: Front Comput Neurosci. 2018 Feb 28;12:11. doi: 10.3389/fncom.2018.00011 (PMC5835533; doi:10.3389/fncom.2018.00011)
Supplement: Table S2 — Early and late modulation in time task, left hemisphere. [file Table2.DOCX]

**Early and late modulation in time task, left hemisphere**

| **Patient** | **Electrode** | **Early modulation**  **(no units)** | **Early normalization factor (μV∙s)** | **Late modulation**  **(no units)** | **Late normalization factor (μV∙s)** |
| --- | --- | --- | --- | --- | --- |
| **1** | LA1 | 0.1966 | 0.0026 | 0.4815 | 0.007 |
|  | LP1 | 0.7267 | 0.002 | 0.3292 | 0.0086 |
|  | LP6 | 0.7899 | 0.0029 | 0.1359 | 0.0048 |
|  | LP7 | 0.1527 | 0.0037 | 0.0378 | 0.0054 |
| **2** | L1 | 0.2212 | 0.0008 | 0.2899 | 0.0066 |
|  | L5 | 1.7408 | 0.0010 | 0.2153 | 0.0030 |
|  | L6 | 1.5949 | 0.0011 | 0.1162 | 0.0036 |
| **3** | LA1 | 0.0047 | 0.0023 | 0.4073 | 0.0024 |
|  | LA2 | 0.1366 | 0.0024 | 0.8495 | 0.0016 |
|  | LP1 | 0.2052 | 0.0025 | 0.2566 | 0.0054 |
|  | LP8 | 1.2191 | 0.0009 | 0.2561 | 0.0008 |
|  | LA8 | 0.5283 | 0.0008 | 0.1628 | 0.001 |
